# A multi-site study on sex differences in cortical thickness in non-demented Parkinson’s disease

**DOI:** 10.1038/s41531-024-00686-2

**Published:** 2024-03-23

**Authors:** Javier Oltra, Barbara Segura, Antonio P. Strafella, Thilo van Eimeren, Naroa Ibarretxe-Bilbao, Maria Diez-Cirarda, Carsten Eggers, Olaia Lucas-Jiménez, Gemma C. Monté-Rubio, Natalia Ojeda, Javier Peña, Marina C. Ruppert, Roser Sala-Llonch, Hendrik Theis, Carme Uribe, Carme Junque

**Affiliations:** 1 Medical Psychology Unit, Department of Medicine, Institute of Neurosciences, University of Barcelona, Faculty of Medicine, Clínic Campus, Carrer de Casanova, 143, Ala Nord, 5th floor, 08036 Barcelona, Catalonia Spain; 2 Institute of Biomedical Research August Pi i Sunyer (IDIBAPS), Carrer del Rosselló, 149, 08036 Barcelona, Catalonia Spain; 3 Centro de Investigación Biomédica en Red Enfermedades Neurodegenerativas (CIBERNED: CB06/05/0018-ISCIII), Hospital Clínic Barcelona, Carrer de Villarroel, 170, 08036 Barcelona, Catalonia Spain; 4 Brain Health Imaging Centre, Centre for Addiction and Mental Health, 250 College St., M5T 1R8 Toronto, ON Canada; 5 Edmond J. Safra Parkinson Disease Program, Neurology Division, Toronto Western Hospital & Krembil Brain Institute, University Health Network, University of Toronto, 399 Bathurst Street, M5T 2S8 Toronto, ON Canada; 6 Multimodal Neuroimaging Group, Department of Nuclear Medicine, University Medical Center Cologne, Kerpener Straße, 62, 50937 Cologne, Germany; 7 Department of Neurology, University Medical Center Cologne, Kerpener Straße, 62, 50937 Cologne, Germany; 8 Department of Psychology, Faculty of Health Sciences, University of Deusto, Avenida de las Universidades, 24, 48007 Bilbao, Basque Country Spain; 9 Department of Neurology, Hospital Clínico San Carlos, Health Research Institute ‘San Carlos’ (IdISCC), Complutense University of Madrid, Calle del Profesor Martín Lagos, s/n, 28040 Madrid, Spain; 10 Department of Neurology, University Hospital of Giessen and Marburg, Center for Mind, Brain and Behavior, University of Marburg and Giessen Universiy, Hans-Meerwein-Straße, 6, 35043 Marburg, Germany; 11 Centre for Comparative Medicine and Bioimaging (CMCiB), Gemans Trias i Pujol Research Institute (IGTP), Camí de les Escoles, s/n, 08916 Badalona, Catalonia Spain; 12 Department of Biomedicine, Institute of Neurosciences, University of Barcelona, Faculty of Medicine, Clínic Campus, Carrer de Casanova, 143, Ala Nord, 5th floor, 08036 Barcelona, Catalonia Spain; 13 Biomedical Imaging Group, Biomedical Research Networking Center in Bioengineering, Biomaterials and Nanomedicine (CIBER-BBN: CB06/01/1039-ISCIII), Carrer de Casanova, 143, 08036 Barcelona, Catalonia Spain

**Keywords:** Parkinson's disease, Diagnostic markers, Neurodegeneration, Translational research

## Abstract

Clinical, cognitive, and atrophy characteristics depending on sex have been previously reported in Parkinson’s disease (PD). However, though sex differences in cortical gray matter measures in early drug naïve patients have been described, little is known about differences in cortical thickness (CTh) as the disease advances. Our multi-site sample comprised 211 non-demented PD patients (64.45% males; mean age 65.58 ± 8.44 years old; mean disease duration 6.42 ± 5.11 years) and 86 healthy controls (50% males; mean age 65.49 ± 9.33 years old) with available T1-weighted 3 T MRI data from four international research centers. Sex differences in regional mean CTh estimations were analyzed using generalized linear models. The relation of CTh in regions showing sex differences with age, disease duration, and age of onset was examined through multiple linear regression. PD males showed thinner cortex than PD females in six frontal (bilateral caudal middle frontal, bilateral superior frontal, left precentral and right pars orbitalis), three parietal (bilateral inferior parietal and left supramarginal), and one limbic region (right posterior cingulate). In PD males, lower CTh values in nine out of ten regions were associated with longer disease duration and older age, whereas in PD females, lower CTh was associated with older age but with longer disease duration only in one region. Overall, male patients show a more widespread pattern of reduced CTh compared with female patients. Disease duration seems more relevant to explain reduced CTh in male patients, suggesting worse prognostic over time. Further studies should explore sex-specific cortical atrophy trajectories using large longitudinal multi-site data.

## Introduction

There is a growing interest in the effect of sex in neurodegenerative diseases. In the precision medicine era, sex differences are relevant to the development of further tailored prevention, diagnosis, and treatment strategies^1,2^. In this context, epidemiology studies report that Parkinson’s disease (PD) is more common in males than females showing a 1.18 overall male/female prevalence ratio according to the most recent metanalysis^3^. Additionally, sex has been spotlighted, in the translational neuroscience field, as a factor with potential influence on different clinical and pathological characteristics of PD^4^.

Regarding the clinical features of PD, the findings point to a distinct pattern depending on sex: a male phenotype characterized by freezing of gait, camptocormia, drooling, worse general cognitive abilities, greater deficits than female non-demented patients in executive functions, and more rapid progression in the severe stage of the disease; and a female phenotype characterized by postural tremor, frequent falls, dysphagia, gastrointestinal dysfunction, pain, and visuospatial impairment^4^. In addition, a different pattern of progression of symptoms between sexes has been revealed by exploring 5-year longitudinal data from 423 PD patients of the Parkinson’s Progression Markers Initiative (PPMI) cohort^5^. The results showed that male patients had a higher decline in motor and non-motor aspects of daily living, showed more progression of motor symptoms assessed in the ON-medication state, and required higher medication dosages over time^5^.

Sex also has been considered a defining feature of neuroimaging phenotypes of several major brain disorders^6^. However, despite the relevance of the research topic, the data on sex differences in gray matter magnetic resonance imaging (MRI) cortical atrophy measures in PD is limited to a few previous studies. First, a single-site study from 2016 analyzed a sample comprised of 43 PD males and 21 PD females with a disease duration of 3.7 years on average through a cortical thickness (CTh) vertex-wise approach^7^. The results showed lower CTh values in male patients compared with female patients in several cortical regions, revealing a mainly parieto-occipital regional pattern of sex differences. Subsequent studies have focused on multicentric MRI data from early drug naïve PD patients of the PPMI cohort^8–10^. In early drug naïve PD patients, deformation-based morphometry (DBM) analyses revealed gray matter atrophy in cortical regions in both directions (PD males > PD females, and vice versa)^8^. Moreover, the authors did not find sex differences in CTh parcellations^8^. In a subsequent study, we found thinner cortex in PD males compared with PD females limited to paracentral and postcentral areas using a vertex-wise procedure^9^. Last, the most recent study using the PPMI cohort explored a large sample of early drug naïve PD patients cross-sectionally and a smaller subsample with two-year follow-ups using vertex-wise CTh and voxel-based morphometry (VBM)^10^. The cross-sectional analyses revealed greater cortical gray matter atrophy mainly in frontal lobe, parietal lobe, and temporal lobe regions in PD males compared with PD females. The longitudinal analyses did not show any significant sex difference in changes over time in those regions that showed sex differences cross-sectionally^10^.

One of the main gaps of the previous studies is that they did not explore the association between regions showing sex differences in gray matter measures and relevant variables that inform of the aging process and the disease course. In this respect, while age contributes to sex differences in regional gray matter cortical reductions in the general population^11,12^, and disease duration^13,14^ and the age of onset^15^ have been associated with cortical gray matter atrophy in PD, their effects on regional sex differences in PD patients have not been explored.

The overall goal of the current study was to investigate sex differences in cortical gray matter atrophy in a large sample of non-demented PD with a wide disease duration range. In this regard, multi-site PD MRI datasets are fundamental to sex differences studies, as a larger sample size protects against statistical power loss due to subgrouping. Our first aim was to characterize sex differences in non-demented PD in CTh, using regional mean CTh measures derived from T1-weighted 3 T MRI acquisitions. We hypothesized that male PD patients would show thinner cortex compared with female PD patients in several regions. Our second aim was to explore the age, disease duration, and age of onset effects on CTh in those regions showing sex differences.

## Results

### Sociodemographic and clinical characteristics

Parkinson’s disease females were older than PD males, and PD males had more years of education than PD females (Table 1). Thus, both variables were included as covariates in further analyses.

**Table 1:** Demographic and clinical characteristics of HC and PD males and females

	HC males *n* = 43	PD males *n* = 136	HC females *n* = 43	PD females *n* = 75	Group effect test stat (*P*-value)	Sex effect test stat (*P*-value)
Age, y	65.14 ± 8.38 (45–81)	64.22 ± 9.41 (40–85)	66.02 ± 8.36 (50–80)	67.80 ± 8.78 (44–86)	0.136 (0.712)	3.688 (0.056)^a^
Education, y	12.93 ± 4.15 (6–23)	11.86 ± 5.08 (0–22)	11.42 ± 4.65 (0–21)	10.52 ± 4.08 (2–20)	2.660 (0.104)	5.583 (0.019)^a^
Disease duration, y	*NA*	7.19 ± 5.03 (1–25)	*NA*	6.94 ± 4.99 (0.7–28)		0.340 (0.734)
Age of onset, y	*NA*	57.03 ± 10.21 (31–78)	*NA*	60.86 ± 8.87 (41–81)		−2.725 (0.007)^a^
MMSE score^b^	29.21 ± 1.29 (24–30)	28.36 ± 1.86 (22–30)	29.38 ± 0.70 (28–30)	28.88 ± 1.10 (26–30)	8.095 (0.005)^c^	2.154 (0.144)
MoCA score^d^	27.11 ± 1.17 (26–29)	25.80 ± 2.97 (17–29)	27.88 ± 1.64 (25–30)	26.25 ± 3.01 (21–30)	193.00 (0.068)	207.00 (0.169)
WMhypo volume, mL	2.53 ± 2.72	2.70 ± 2.53	2.57 ± 1.85	2.87 ± 2.32	0.580 (0.447)	0.115 (0.735)
High Fazekas score presence, *n* (%)	5 (11.6%)	10 (13.2%)	5 (11.6%)	10 (13.3%)	0.148 (0.701)	0.001 (0.972)
eTIV, mL	1536.73 ± 150.96	1574.14 ± 155.74	1374.68 ± 132.77	1377.26 ± 156.92	1.027 (0.312)	82.679 ( < 0.001)^e,f^

Data are presented by groups as mean ± SD, followed by range as (minimum-maximum). For volumetric variables, WM-hypo volume and eTIV data are presented by groups as mean ± SD. Two-way analysis of variance (ANOVA) followed by the LSD tests was used for all demographic variables, cognitive, and volumetric variables; except for MoCA score, for which Mann–Whitney *U* was used. Independent samples *t*-test was used for all clinical variables. Fazekas score, dichotomized as low or high score, was analyzed using chi-squared tests. *eTIV* estimated total intracranial volume; *HC* healthy controls; *MMSE* Mini-Mental State Examination; *MoCA* Montreal Cognitive Assessment; *NA* not applicable; *PD* Parkinson’s disease; *SD* standard deviation; *stat* statistic; *WM-hypo* white matter hypointensities; *y* years.

^a^Sex differences in the PD group (*P*-value ≤ 0.05).

^b^MMSE score was available for 29 HC males, 72 PD males, 26 HC females, and 50 PD females.

^c^Differences between HC males and PD males (*P*-value < 0.01).

^d^MoCA scores were available for 9 HC males, 25 PD males, 8 HC females, and 8 PD females.

^e^Sex differences in the HC group (*P*-value < 0.001).

^f^Sex differences in the PD group (*P*-value < 0.001).

Concerning clinical variables, PD females had a statistically significant older age of onset than PD males (Table 1). Therefore, it was included as a covariate when comparing between sexes within the PD group.

Regarding global cognitive scores, PD males had statistically significantly lower scores than healthy control (HC) males in Mini-Mental State Examination (MMSE, Table 1).

Regarding MRI variables, there were no significant differences in WMH-hypo volume nor in the presence of high Fazekas scores. As expected, the estimated total intracranial volume (eTIV) was higher in males than in females in both PD and HC groups (Table 1).

### Sex differences in cortical thickness in Parkinson’s disease patients

First, the results of the GLM, including age and years of education as covariates, revealed statistically significant group by sex interaction in the left and right hemisphere mean CTh estimations, as well as in mean CTh from seven frontal, five temporal, nine parietal, six occipital, and two limbic cortical regions (Supplementary Table 1). Subsequent comparisons were circumscribed to these regions.

**Fig. 1:**
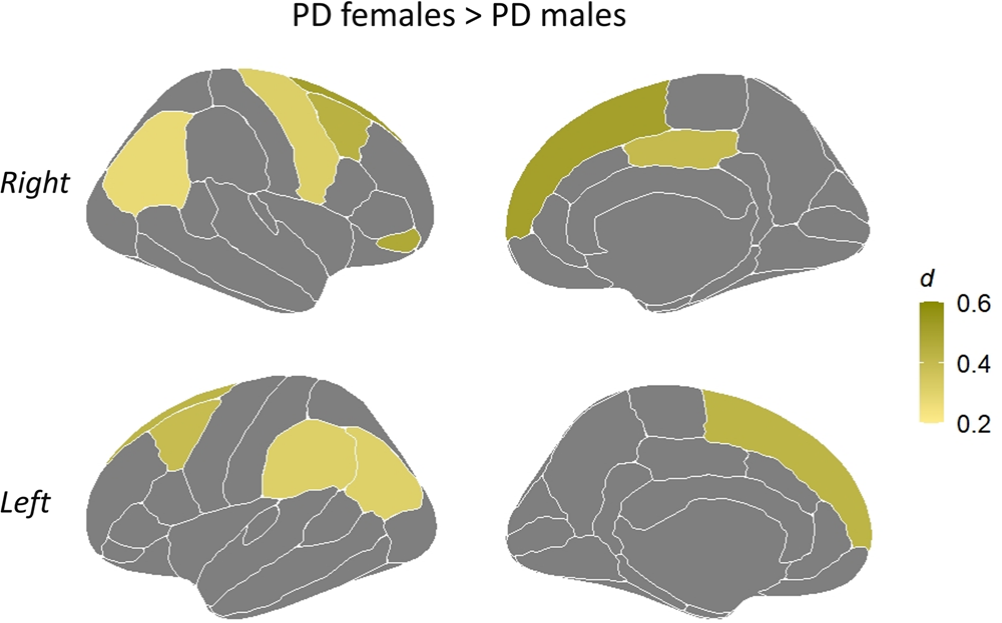
Statistically significant thinner regions in PD males compared with PD females from comparisons including the age of onset as an extra covariate (FDR adjusted *P*-value ≤ 0.05). The color bar indicates the Cohen’s *d* effect size for each region. PD Parkinson’s disease. Regions represented correspond to the 68 cortical brain regions of the Desikan–Killiany atlas^34^. The list of regions showing statistically significant results is available in Supplementary Table 2.

Between sex comparisons within the PD group, including age and education as covariates plus age of onset as an extra covariate, showed lower CTh values in PD males compared with PD females in the right mean estimation, as well as in six frontal (bilateral caudal middle frontal, bilateral superior frontal, right precentral, and right pars orbitalis), three parietal (bilateral inferior parietal and left supramarginal) and one limbic regions (right posterior cingulate) (Fig. 1, Supplementary Table 2). There were no statistically significant differences in the opposite direction. Comparisons between sexes within the HC group did not reveal any statistically significant difference.

The supplementary analysis revealed that there were no statistically significant differences between PD males and PD females in the brain parenchymal fraction (BPF; PD males, *M* = 0.717, *SD* = 0.041; PD females, *M* = 0.720, *SD* = 0.048; *t* = 0.472, *P*-value = 0.637) and all sex differences within the PD group (i.e., for the right mean CTh and the ten regional estimations) remained statistically significant after including the BPF as an extra covariate (Supplementary Table 3).

**Fig. 2:**
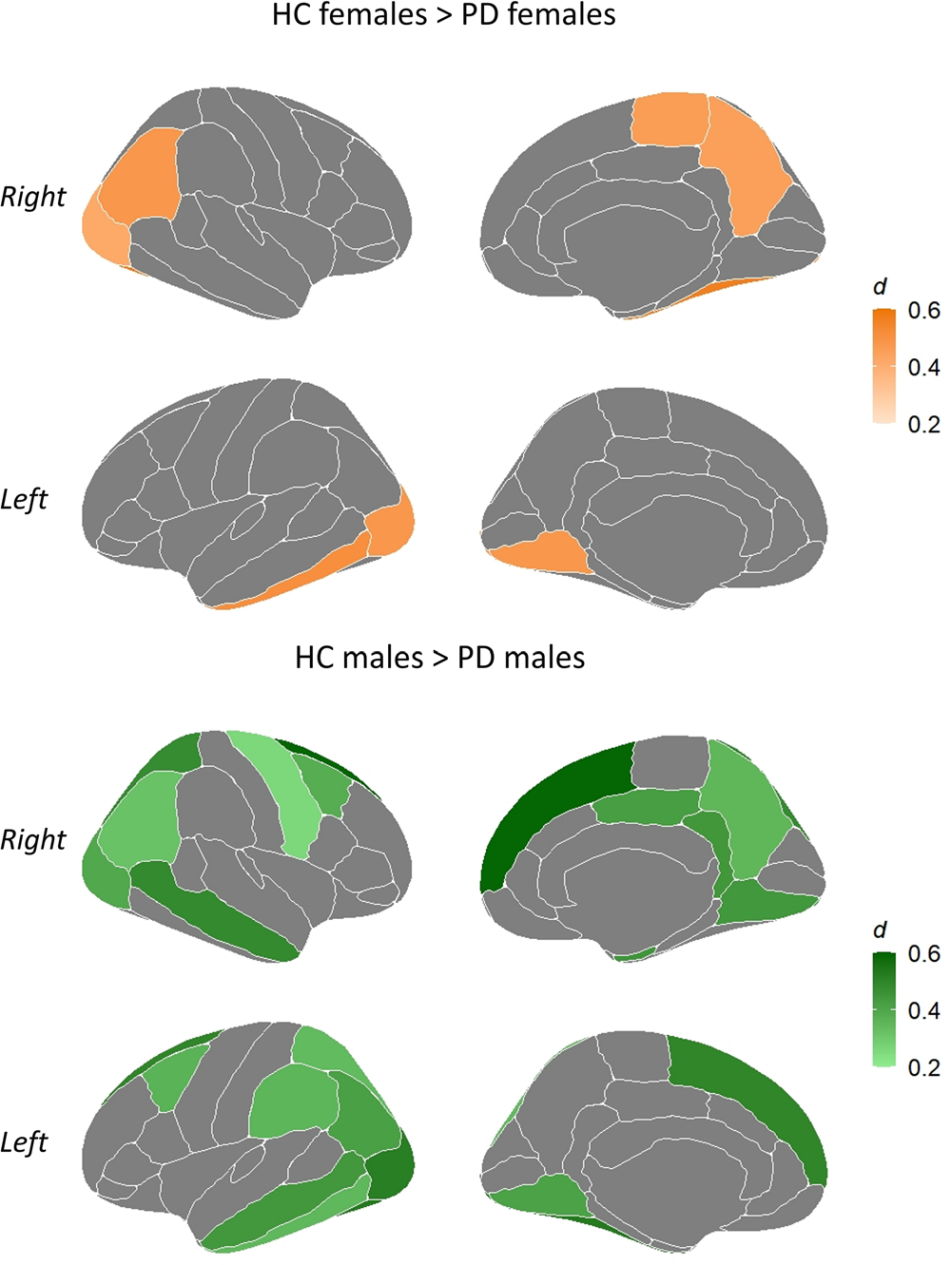
Statistically significant thinner regions in PD females compared with HC females and in PD males compared with HC males (FDR adjusted *P*-value ≤ 0.05). The color bars indicate the Cohen’s *d* effect size for each region. HC healthy controls; PD Parkinson’s disease. Regions represented correspond to the 68 cortical brain regions of the Desikan-Killiany atlas^34^. The list of regions showing statistically significant results is available in Supplementary Table 1.

When comparing PD patients with HC by sexes, including age and years of education as covariates, we found that PD females showed thinner cortex in one temporal (left inferior temporal), three parietal (right inferior parietal, right paracentral, and right precuneus), and four occipital regions (bilateral lateral occipital, left lingual, and right fusiform) compared with HC females (Fig. 2 and Supplementary Table 1). On the other hand, PD males compared with HC males showed lower CTh values in the right mean estimation and five frontal (bilateral caudal middle frontal, bilateral superior frontal, and right precentral), four temporal (bilateral middle temporal, left inferior temporal, and right entorhinal), six parietal (bilateral inferior parietal, bilateral superior parietal, left supramarginal, and right precuneus), six occipital (bilateral fusiform, bilateral lateral occipital, and bilateral lingual), and two limbic regions (right isthmus and right posterior cingulate). There were no statistically significant differences in the opposite direction (Fig. 2 and Supplementary Table 1).

### Age, disease duration, and age of onset as explanatory variables of regional cortical thickness

In PD males, age and disease duration were negatively associated with CTh, for nine out of ten regions, except for one region in which only age was associated with CTh (Tables 2 and 3). Thus, older age together with longer disease duration was significantly associated with thinner cortex in PD males in almost all regions showing sex differences. On the other hand, in PD females, age was negatively associated with thinner cortex in eight out of ten regions, whereas longer disease duration was associated with thinner cortex only in one region (Tables 2 and 3).

**Table 2:** Resulting models from regression analyses including disease duration for prediction of mean cortical thickness in regions showing sex differences

	PD males		PD females	
	Variables	*t*-stat (*P*-value)	Variables	*t*-stat (*P*-value)
L caudal middle frontal	Age	**−2.740 (0.007)**	Age	**−2.689 (0.009)**
	Disease duration	**−2.678 (0.008)**		
	Education	−1.949 (0.053)		
L superior frontal	Age	**−3.174 (0.002)**	Age	**−3.103 (0.003)**
	Disease duration	**−2.346 (0.020)**	Education	−1.431 (0.156)
R caudal middle frontal	Age	**−2.494 (0.014)**	Non-significant model	
	Disease duration	−1.811 (0.072)		
R pars orbitalis	Age	**−2.216 (0.028)**	Disease duration	**−2.495 (0.015)**
	Disease duration	**−2.149 (0.034)**	Education	−1.400 (0.166)
	Education	−1.741 (0.084**)**		
R precentral	Age	**−5.417 (**<**0.001)**	Age	**−2.493 (0.015)**
	Disease duration	**−2.164 (0.032)**		
R superior frontal	Age	**−2.004 (0.047)**	Age	**−2.751 (0.008)**
	Disease duration	**−2.242 (0.027)**	Education	**−2.291 (0.025)**
L inferior parietal	Age	**−4.485 (**<**0.001)**	Age	**−2.747 (0.008)**
	Disease duration	**−3.486 (**<**0.001)**	Disease duration	−1.638 (0.106)
			Education	−1.652 (0.103)
L supramarginal	Age	**−4.824 (**<**0.001)**	Age	**−3.190 (0.002)**
	Disease duration	**−2.678 (0.008)**		
R inferior parietal	Age	**−5.683 (**<**0.001)**	Age	**−3.217 (0.002)**
	Disease duration	**−3.659 (**<**0.001)**	Disease duration	−1.738 (0.087)
R posterior cingulate	Age	**−3.122 (0.002)**	Age	**−2.396 (0.019)**
	Disease duration	**−2.214 (0.029)**	Disease duration	−1.592 (0.116)

Linear regression analyses with stepwise selection based on the Akaike information criterion (AIC) were applied separately in male and female patients for each region showing sex differences (response variable) with age, disease duration, and years of education introduced as explanatory variables. The selected variables for each model are shown, with statistically significant response variables in bold (*P*-value ≤ 0.05). *L* left; *PD* Parkinson’s disease; *R* right; stat statistic.

The selected variables for each model are shown, with statistically significant response variables in bold (*P*-value ≤ 0.05).

**Table 3:** Goodness of fit of the resulting models from regression analyses, including disease duration for prediction of mean cortical thickness in regions showing sex differences

	PD males			PD females		
	Adjusted R^2^	*F*	*P*-value	Adjusted R^2^	*F*	*P*-value
L caudal middle frontal	0.088	5.345	0.002	0.078	7.233	0.009
L superior frontal	0.102	8.639	≤0.001	0.094	4.833	0.011
R caudal middle frontal	0.059	5.269	0.006	Non-significant model		
R pars orbitalis	0.054	3.551	0.016	0.076	4.040	0.023
R precentral	0.205	18.410	≤0.001	0.066	6.213	0.015
R superior frontal	0.056	5.033	0.008	0.089	4.617	0.013
L inferior parietal	0.200	17.920	≤0.001	0.131	4.715	0.005
L supramarginal	0.189	16.720	≤0.001	0.110	10.180	0.002
R inferior parietal	0.264	25.230	≤0.001	0.174	8.773	≤ 0.001
R posterior cingulate	0.095	8.117	≤0.001	0.109	5.528	0.006

Linear regression analyses with stepwise selection based on the Akaike information criterion (AIC) were applied separately in male and female patients for each region showing sex differences (response variable) with age, disease duration, and years of education introduced as explanatory variables. The goodness of fit, statistics, and statistical significance for each resulting model are shown. *L* left; *PD* Parkinson’s disease; *R* right.

Complementary, as expected, the analyses that included age of onset as an explanatory variable instead of disease duration showed congruent results, revealing significant associations of older age and younger age of onset with thinner cortex in nine out of ten regions in male patients, but only in one region in female patients (Supplementary Tables 4 and 5).

Altogether, older age, longer disease duration, and younger age of onset significantly contributed to explaining reduced CTh in PD males in regions showing sex differences.

### Sex differences in subcortical gray matter volume in Parkinson’s disease patients

Our supplementary analyses showed higher gray matter volume in the brainstem, bilateral thalamus, and right amygdala in PD males compared with PD females (Supplementary Table 6). After excluding three cases of male patients displaying extreme values for the brainstem and the thalamus (*z*-score ≥ 3), only sex differences for the right amygdala remained significant in the PD group, which was observed in the HC group in the same direction.

## Discussion

In this study, the main aim was to analyze sex differences in regional mean CTh estimations in a large sample of non-demented PD using a multi-site approach. We found that male patients had thinner cortex in several regions compared with female patients. Furthermore, for those regions showing a significant group by sex interaction, the pattern of reduced CTh in patients compared with controls involved more areas in male patients than in female patients. Whereas the pattern of reduced CTh in female patients was more restricted to posterior regions, the male patients had a more extended pattern of thinner cortical regions, including frontal, parietal, and limbic regions. On the other hand, aging and disease course-related variables (disease duration and age of onset) contributed to explaining reduced CTh differently in male and female PD patients in regions showing sex differences.

In the current study, the between-sexes comparisons revealed that male PD patients had thinner cortex compared with female PD patients in several brain regions, including six frontal (bilateral caudal middle frontal, bilateral superior frontal, right precentral, and right pars orbitalis), three parietal (bilateral inferior parietal and left supramarginal), and one limbic (right posterior cingulate). These findings partially agree with a previous report that showed widespread reduced CTh in several areas in male PD patients compared with female PD patients with a disease duration of 3.7 years on average using a vertex-wise approach^7^. Of note, the results from the previous study also involved bilateral caudal middle frontal, bilateral superior frontal, bilateral inferior parietal, and left supramarginal regions^7^. However, the authors analyzed single-site 1.5 T MRI data from 64 PD patients (43 males and 21 females) and 46 HC (12 males and 34 females). Thus, even though the effect sizes were not reported, the reduced sizes of the subgroups could mitigate the statistical power, especially for PD females and HC males. It might partially explain the absence of significant differences in other comparisons; for example, between male patients and controls^7^. Furthermore, they did not explore the group by sex interaction, which also limits the interpretation of the findings. In this respect, we found that male patients had reduced CTh compared with male controls in almost all the regions showing sex differences within the PD group (nine out of ten). Moreover, we previously guaranteed that there were significant group by sex interactions for all regions in which sex differences were explored.

Our results showed sex differences in CTh in PD, revealing several thinner regions in male patients compared with female patients. These findings contrast with previous results from early drug naïve PD patients from the PPMI cohort^8–10^. In this respect, analyses on CTh parcellations did not show differences in a previous study^8^. In a subsequent study, we explored sex differences by applying a CTh vertex-wise approach^9^. We found that male patients showed thinner cortex in small clusters limited to left postcentral and right precentral areas. However, the comparison between male patients and controls did not report differences in these regions^9^. Finally, the most recent study exploring early drug naïve patients from this cohort found more widespread reduced CTh in PD males than PD females, including frontal, parietal, and temporal areas, using a vertex-wise approach^10^. In line with our results, the authors found thinner cortex in the right precentral and left cingulate areas in PD males compared with PD females^10^. As a matter of interest, the three early drug naïve PD samples came from the same time point of the PPMI cohort, and probably the samples were partially overlapped. Altogether, previous and current findings suggest that sex differences are present in early drug naïve PD patients, showing mainly a more marked pattern of reduced CTh in male patients; this pattern might be more pronounced when the disease duration is longer. These findings might reflect that the cortical gray matter atrophy trajectories differ between male and female patients. However, the only longitudinal exploration to date revealed no significant sex differences in the gray matter atrophy longitudinal changes in early drug naïve PD patients after 2-year follow-ups analyzing a subgroup of 66 PD males and 31 PD females from the PPMI cohort^10^. Further studies should address if there are sex-specific gray matter atrophy trajectories using larger multi-site data and several time points, ideally from preclinical stages and with long-term follow-ups.

Lower CTh estimations in PD males compared with PD females were concentrated mainly on right hemisphere regions in contrast with left hemisphere regions (i.e., six in the right versus four in the left hemisphere; see Fig. 1 and Supplementary Table 2). In the same direction, the global measure of right mean CTh was lower in PD males than in PD females. One could argue the existence of a lateralization pattern of cortical atrophy depending on sex. Interestingly, previous studies have reported predominant left-hemispheric findings in PD^16–18^. Then, one could hypothesize that male and female patients have similar atrophy rates in some left-hemispheric regions, with a more pronounced right-hemispheric atrophy in male patients. However, the lower number of findings in the left hemisphere does not necessarily imply a right atrophy preponderance in male patients. In this regard, previous studies focused on sex differences in gray matter measures in PD did not support the existence of a lateralized pattern of sex differences. Moreover, healthy population-based studies did not report lateralized sex differences in gray matter measures either^11,19^. Furthermore, the used approach (e.g., regional mean cortical thickness instead of vertex-wise) and specific threshold established for statistical significance could contribute to a slight lateralization of results in our study.

Next, we explored the relationship between CTh in regions showing sex differences in PD and aging and disease course-related variables. Regarding PD males, older age and longer disease duration were significantly associated with thinner cortex in almost all the regions that previously showed sex differences. Instead, in PD female patients, older age but no longer disease duration was associated with thinner cortex in these regions. In accordance, the same effect was observed when introducing age of onset in the regression analyses. It could be hypothesized that female patients may show cortical thinning trajectories close to normal aging in certain areas. Whilst male patients show a stepper decline along disease duration in these areas. These findings reinforce the relevance of exploring longitudinal trajectories to reveal if PD patients display different cortical atrophy trajectories depending on sex.

Our supplementary analyses showed lower gray matter volume in the right amygdala in PD females compared with PD males, also observed in the HC group. This pattern of sex differences in the amygdala has been reported previously in population-based studies in adults; however, metanalytical results show reduced size effects not supporting a sex dimorphism of this structure^20^. Therefore, this regional finding may not reflect disease-dependent sex differences in our sample. The absence of sex differences in subcortical gray matter volumes showing lower estimations in PD males contrasts with previous findings^8,9,21^. Notably, our study sample is heterogeneous for disease duration, contrasting with the de novo PD samples used in the prior reports^8,9,21^. Moreover, a recent longitudinal study did not show a widespread pattern of lower subcortical gray matter volume in male patients at follow-up, limiting the findings to the bilateral cerebellum crus (I and II lobules)^10^.

The main strength of the current report was the use of cortical gray matter MRI data from a large multi-site sample from four international research centers applying a harmonization approach which guarantees better control of the site effect^22^. The sampling strategy is relevant since the female group size in PD samples is frequently smaller in single-site MRI studies, which does not allow for performing sex differences analyses. Furthermore, prior explorations of sex differences using data from multiple centers did not control for the multi-site bias, even when combining 1.5 and 3 Tesla acquisitions^8,9^. The main limitation of the study was that the data was limited to cross-sectional MRI acquisitions. In this regard, future research should address sex differences in cortical atrophy trajectories using longitudinal MRI data from preclinical to advanced stages and with long-term follow-ups. Moreover, clinical data, biomarkers of co-pathology, and genetic variants were not systematically collected in the four research centers, which limited further analyses focused on the association of sex with other variables apart from those explored in this study (i.e., age, disease duration, and age of onset). Incorporating standardized protocols may enhance the characterization of multi-site samples for research purposes. Future studies should cover if sex-dependent atrophy trajectories correlate with clinical and cognitive outcomes. Besides, further research should address whether patterns of neurodegeneration vary depending on sex within the previously described longitudinal clinical subtypes and cross-sectional atrophy subtypes of the disease^23–27^, considering also reported sex differences in genetic factors in PD^28^. Another limitation of our study is the absence of a standardized protocol for neuropsychological assessment. For a better characterization of cognitive profile in PD multi-site studies, further initiatives are encouraged to use harmonized protocols^29^.

Further studies should cover the impact of cerebrovascular disease and Alzheimer’s disease co-pathologies on sex differences in PD. For example, we have included WMH-hypo volume extracted using FreeSurfer as a control variable; however, further studies should address the impact of WMH-hypo/WMH-hyper on the effects of sex in PD using advanced lesion segmentation techniques (i.e., Bayesian models and deep learning approaches).

Evidence on sex differences in gray matter atrophy markers is relevant to developing diagnostic and prognostic approaches in the context of personalized medicine. Parallel to these advances, the efforts need to focus on the mechanisms behind the observed differences, from the neuroprotective role of estrogens^30^ to genetics^28^. In this sense, there is no established model about how sex impacts PD. From this perspective, in a recent review, Cerri et al. pointed out three levels in which sex influences PD pathophysiology differentially: dopaminergic neurodegeneration, neuroinflammation, and oxidative stress^4^.

In conclusion, non-demented male PD patients show increased cortical gray matter atrophy compared with female patients, displaying thinner cortex in several regions involving all the cortical lobes. Cortical thickness in regions showing sex differences seems explained by disease duration only for male patients. Further studies should address whether PD patients show different cortical atrophy trajectories depending on sex, which is relevant for the advancement of precision medicine approaches.

## Methods

### Participants

We used multi-site MRI data from four research centers: the University of Deusto (Bilbao, Spain; Site 1), the University of Barcelona (Barcelona, Spain; Site 2), the Center of Addiction and Mental Health (CAMH; Toronto, Canada; Site 3), and the University of Cologne (Cologne, Germany; Site 4). The initial sample comprised 216 PD and 87 HC individuals, previously described in Monté-Rubio et al.^31^. The PD patients included in this sample fulfilled the UK PD Society Brain Bank diagnostic criteria for PD and were classified as non-demented according to the Level I for PD dementia diagnosis from the Movement Disorder Society Task Force on Dementia in Parkinson’s Disease^32,33^. After preprocessing, we excluded 5 PD and 1 HC due to segmentation problems; see the MRI preprocessing section below. Thus, our final sample comprised 211 PD and 86 HC volunteers: 136 PD males, 75 PD females, 43 HC males, and 43 HC females. The distribution of participants per center, including division by group and sex, is shown in Supplementary Table 7.

We considered age, sex, and years of education as demographic variables. The clinical variables included disease duration in years, age of onset, and global cognitive scores (i.e., MMSE; Montreal Cognitive Assessment, MoCA).

All participating sites received approval from an ethical standards committee prior to study initiation (i.e., Site 1, Clinical Research Ethics Committee of the Basque Country and Ethics Committee of the University of Deusto; Site 2, Ethics Committee of the University of Barcelona; Site 3, Ethics Committee of the Center for Addiction and Mental Health; Site 4, Ethics Committee of the Medical Faculty of the University of Cologne), the study was conducted according to the guidelines of the Declaration of Helsinki, and all sites obtained written informed consent for research from all participants in the study.

### MRI acquisition

All participants have available T1-weighted MRI data acquired using 3 T scanners. The characteristics of the acquisition parameters for the MRI images are described below:

Site 1: An MRI scanner Philips Achieva 3 T TX was used to obtain the images in a sagittal orientation. Repetition time (TR) = 7.4 ms, echo time (TE) = 3.4 ms, matrix size 228 × 218 mm^2^, flip angle 9°, field of view (FOV) = 250 mm, slice thickness 1.1 mm, acquisition time = 4′55″, 300 slices, voxel size 0.98 × 0.98 × 0.60 mm^3^.

Site 2: An 8-channel head coil SIEMENS MAGNETOM TrioTim syngo MR B19 3 T scanner (Siemens) was used to obtain high-resolution three-dimensional (3D) T1-weighted images in a sagittal orientation. TR = 2300 ms, TE = 2.98 ms, matrix size = 256 × 256 mm^2^, flip angle 9°, FOV = 256 mm, acquisition time = 7′48″, 240 slices, voxel size 1.0 × 1.0 × 1.0 mm^3^.

Site 3: Images were acquired through a General Electric Discovery MR750 3 T scanner, using a fast-spoiled gradient echo pulse sequence in a sagittal orientation. TR = 6.7 ms, TE = 3.0 ms, matrix size 256 × 256 mm^2^, flip angle 8°, FOV = 230 mm, acquisition time = 4′16″, 200 slices, voxel size 0.89 × 0.89 × 0.9 mm^3^.

Site 4: A PRISMA MAGNETOM 3 T scanner (Siemens) was used to obtain T1-weighted images in a sagittal orientation. TR = 2.300 ms, TE = 2.32 ms, matrix size 256 × 256 mm^2^, flip angle = 8°, FOV = 230 mm, acquisition time = 5′30″, 192 slices, voxel size 0.9 × 0.9 × 0.9 mm^3^.

### MRI preprocessing

Regional mean CTh estimations of the 68 cortical brain regions of the Desikan–Killiany atlas^34^ and left and right hemisphere mean CTh estimations were extracted after applying the automated processing pipeline and FreeSurfer v6.0.0 tools (https://surfer.nmr.mgh.harvard.edu/). The stream includes the parcellation of the cerebral cortex and its automated labeling^34,35^. After preprocessing, data generated for all subjects were visually inspected to guarantee the accuracy of registration, skull stripping, segmentation, and cortical surface reconstruction. Furthermore, possible errors were fixed using standard procedures. After this step, we excluded 5 PD and 1 HC due to segmentation problems. Thus, our final sample comprised 211 PD and 86 HC.

We extracted white matter hypointensities (WM-hypo) volume as a proxy of small vessel disease after applying a probabilistic labeling implementation in FreeSurfer v6.0.0 to T1-weighted images. As a note, WM-hypo is strongly correlated with the unidentified bright objects on T2/FLAIR sequences, generally referred to as white matter hyperintensities (WMH-hyper)^36^. Then, we classified participants into low and high Fazekas scores by applying an established cut-off^36^. Since WM-hypo could cause errors in FreeSurfer gray matter segmentation^37^, we visually reinspected the outputs for those participants showing a high Fazekas score to ensure its quality. After this second quality check, we did not make additional exclusions.

### Harmonization of automated regional mean cortical thickness estimations

We applied the prior validated ComBat method to harmonize regional mean CTh values across sites^22^ using its implementation in Matlab R2020b^38^ and the parametric empirical Bayes framework. This technique, adapted from the genomics research field^39^, allows handling with non-biological variance due to the use of different MRI scanners and acquisition protocols^22^. Thus, we applied this procedure to the 70 mean CTh measures considering the four centers. Furthermore, we included group (PD or HC), sex, age, and years of education as sources of variance to be preserved.

### Statistical analyses

Group and sex effects in sociodemographic variables were analyzed through two-way analysis of variance (ANOVA) models followed by least significant difference (LSD) tests. Sex differences in clinical variables were analyzed by independent samples *t*-tests. These analyses were performed using SPSS version 27.0^40^.

We applied generalized linear models (GLM) with Monte Carlo permutation tests with 999 iterations using in-house methods written in Matlab R2020b^38^ to analyze group, sex, and group by sex interaction effects for the 68 regional and left and right automated mean CTh harmonized estimations. Then, we performed post hoc pairwise comparisons only for those regions showing statistically significant interactions, also using GLM models and Monte Carlo permutation tests with 999 iterations. Sociodemographic variables were considered covariates in these models as required, and an equivalent GLM approach was applied, controlling for clinical covariates in the PD group (i.e., age, years of education, and age of onset, respectively; please see above in *Results: Sociodemographic and clinical characteristics*). Cohen’s *d* effect sizes^41^ were computed for post hoc comparisons using JASP version 0.14.3^42^. To control the rate of type I errors, the false discovery rate (FDR) approach, through the Benjamini-Hochberg procedure, was implemented for each contrast^43,44^. For data visualization, the “ggseg” package was used to plot the statistically significant differences in mean CTh estimations^45^ using R version 4.1.2^46^ with the RStudio version 2022.02.0 interface^47^.

Multiple linear regression analyses were conducted to explore the age, disease duration, and age of onset effects on regions showing sex differences in PD. As a response variable, each model included a mean CTh estimation showing statistically significant sex differences in PD and age, plus disease duration or age of onset as explanatory variables, as well as the sociodemographic covariates introduced in the previous GLM models (i.e., years of education). The disease duration and age of onset effects were explored using two separate models to avoid multicollinearity; note that disease duration was derived by subtracting the age of onset from age. We tested the models separately for PD male and PD female patients. A stepwise model selection procedure by the Akaike information criterion (AIC) was applied to select the best-fitted model^48^. Multiple linear regression analyses were also performed using R version 4.1.2^46^ with the RStudio version 2022.02.0 interface^47^.

As supplementary analyses, we included equivalent GLM models with subcortical gray matter volumes extracted using FreeSurfer v6.0.0 as dependent variables (i.e., thalamus, putamen, pallidum, caudate, hippocampus, amygdala, accumbens, and brainstem). We harmonized the measures using ComBat as described previously and adjusted them for eTIV by applying a residual approach^49,50^. The eTIV volume was extracted and harmonized using the same procedures explained above. Additionally, to clarify whether the observed sex differences in CTh in PD were influenced by the whole brain atrophy, as a proposed proxy of brain reserve, we used the BPF^51,52^. We computed BPF as the ratio of gray matter plus white matter, excluding ventricles (i.e., *BrainSegNotVent* from Freesurfer) to eTIV. We included the BPF as an extra covariate in a GLM model for post hoc pairwise comparisons between PD males and PD females (i.e., controlling for age, education, age of onset, and BPF). We specifically examined those CTh measures in which there were statistically significant differences in the main post hoc analysis (i.e., with age, education, and age of onset as covariates).

For all analyses, the statistical significance threshold was set at a two-tailed *P*-value ≤ 0.05.

### Supplementary information

Supplementary Material

Related Manuscript File

## Data Availability

The de-identified data that support the findings of this study are available on request from the corresponding author (BS) under data sharing agreement and approval for the appropriate ethics committees. The data are not publicly available due to privacy or ethical restrictions.
