# Supplementary material for: A multi-site study on sex differences in cortical thickness in non-demented Parkinson’s disease
Source: NPJ Parkinsons Dis. 2024 Mar 23;10:69. doi: 10.1038/s41531-024-00686-2 (PMC10960793; doi:10.1038/s41531-024-00686-2)
Supplement: Supplementary file 1 — Supplementary Material [file 41531_2024_686_MOESM1_ESM.pdf]

Supplementary Table 1 Regions showing significant group by sex interactions in cortical thickness parcellations

	HC males	PD males	HC females	PD females	*F*	P^{G}	P^{S}	P^{GS}	HC males > PD males			HC females > PD females			PD females > PD males		
									t	*P*-value	*d*	*t*	*P*-value	*d*	*t*	*P*-value	*d*
Global																	
L mean thickness	2.44±0.09	2.40±0.11	2.45±0.10	2.41±0.11	5.371	0.006	0.011	0.011	2.641	0.019	0.420		—			—	
R mean thickness	2.45±0.09	2.40±0.10	2.45±0.10	2.42±0.11	6.758	0.006	0.011	0.009	3.133	0.016	0.502		—		2.307	0.022	0.308
Frontal																	
L caudal middle frontal	2.50±0.12	2.46±0.14	2.53±0.14	2.51±0.15	5.228	0.006	0.011	0.011	2.127	0.023	0.364		—		2.957	0.010	0.419
L precentral	2.54±0.11	2.51±0.15	2.59±0.14	2.54±0.16	5.071	0.012	0.011	0.016		—			—		2.055	0.038	0.281
L superior frontal	2.66±0.15	2.60±0.13	2.66±0.15	2.64±0.13	6.160	0.006	0.011	0.009	2.924	0.016	0.495		—		3.153	0.010	0.444
R caudal middle frontal	2.48±0.15	2.43±0.14	2.48±0.14	2.49±0.15	4.356	0.015	0.011	0.019	2.197	0.023	0.378		—		3.151	0.008	0.457
R pars orbitalis	2.57±0.17	2.54±0.17	2.61±0.15	2.61±0.16	4.648	0.015	0.013	0.019		—			—		3.451	0.008	0.498
R precentral	2.49±0.11	2.46±0.17	2.55±0.13	2.49±0.16	5.966	0.010	0.011	0.009	1.637	0.050	0.260		—		2.440	0.021	0.333
R superior frontal	2.63±0.14	2.56±0.13	2.63±0.13	2.62±0.14	7.588	0.006	0.011	0.009	3.400	0.016	0.592		—		3.726	0.008	0.534
Temporal																	
L inferior temporal	2.76±0.15	2.71±0.16	2.76±0.14	2.68±0.14	3.706	0.034	0.028	0.036	2.087	0.025	0.349	2.783	0.040	0.510		—	
L middle temporal	2.79±0.13	2.73±0.16	2.76±0.15	2.70±0.16	3.554	0.034	0.038	0.043	2.804	0.017	0.441		—			—	
L transverse temporal	2.27±0.21	2.25±0.23	2.36±0.19	2.28±0.22	3.357	0.035	0.039	0.043		—			—			—	
R entorhinal	3.63±0.37	3.50±0.34	3.62±0.36	3.52±0.37	3.792	0.034	0.036	0.022	2.742	0.017	0.448		—			—	
R middle temporal	2.84±0.13	2.77±0.15	2.81±0.11	2.78±0.15	4.116	0.023	0.016	0.022	3.018	0.016	0.484		—			—	
Parietal																	
L inferior parietal	2.38±0.14	2.33±0.14	2.40±0.16	2.36±0.14	5.788	0.006	0.011	0.011	2.567	0.019	0.418		—		2.471	0.018	0.340
L postcentral	2.04±0.11	2.03±0.13	2.09±0.11	2.05±0.13	3.639	0.032	0.032	0.036		—			—		1.871	0.050	0.260
L precuneus	2.32±0.11	2.30±0.14	2.36±0.15	2.30±0.15	3.268	0.044	0.057	0.050		—			—			—	
L superior parietal	2.17±0.14	2.13±0.l4	2.20±0.14	2.15±0.14	4.524	0.012	0.011	0.019	1.992	0.034	0.342		—			—	
L supramarginal	2.47±0.13	2.43±0.14	2.49±0.14	2.46±0.15	4.524	0.012	0.011	0.019	2.194	0.023	0.355		—		2.426	0.019	0.330
R inferior parietal	2.42±0.13	2.39±0.14	2.49±0.15	2.41±0.15	8.674	0.006	0.011	0.009	2.057	0.030	0.323	2.744	0.040	0.480	2.295	0.025	0.304
R paracentral	2.39±0.14	2.37±0.14	2.45±0.14	2.38±0.15	4.535	0.015	0.011	0.016		—		2.524	0.040	0.461		—	
R precuneus	2.35±0.13	2.30±0.15	2.38±0.15	2.31±0.14	5.772	0.010	0.011	0.011	2.209	0.025	0.354	2.575	0.040	0.449		—	
R superior parietal	2.18±0.12	2.12±0.14	2.20±0.15	2.13±0.14	5.656	0.010	0.011	0.011	2.823	0.017	0.478		—			—	
Occipital																	
L fusiform	2.72±0.14	2.65±0.14	2.72±0.15	2.66±0.13	6.231	0.006	0.011	0.009	3.228	0.016	0.527		—			—	
L lateral occipital	2.18±0.13	2.12±0.13	2.21±0.14	2.14±0.12	7.944	0.006	0.011	0.009	3.016	0.016	0.516	2.619	0.040	0.478	1.921	0.038	0.270
L lingual	2.01±0.13	1.96±0.11	2.00±0.12	1.94±0.10	4.126	0.015	0.021	0.019	2.504	0.019	0.413	2.562	0.040	0.481		—	
R fusiform	2.76±0.15	2.67±0.15	2.76±0.14	2.66±0.14	9.336	0.006	0.011	0.009	3.888	0.016	0.619	3.235	0.031	0.565		—	
R lateral occipital	2.26±0.14	2.21±0.14	2.28±0.14	2.21±0.14	4.499	0.015	0.011	0.016	2.352	0.023	0.393	2.291	0.043	0.419		—	
R lingual	2.04±0.13	1.99±0.10	2.04±0.13	1.98±0.12	4.078	0.015	0.023	0.022	2.595	0.019	0.443		—			—	
Limbic																	
R isthmus cingulate	2.40±0.20	2.31±0.21	2.37±0.22	2.34±0.23	3.402	0.031	0.049	0.050	2.667	0.016	0.444		—		1.983	0.036	0.277
R posterior cingulate	2.45±0.15	2.38±0.16	2.46±0.17	2.43±0.17	5.225	0.006	0.011	0.016	2.518	0.019	0.425		—		3.002	0.008	0.422

Cortical thickness data in mm are presented by groups as mean ± SD. General linear model including age and years of education as covariates and Monte Carlo permutation testing with 999 iterations followed by FDR correction (*P*-value ≤ 0.05) was used to compute group, sex, group by sex interaction effects, and post hoc pairwise comparisons. Abbreviations: *FDR*, false discovery rate; *HC*, healthy controls; *L*, left; *PD*, Parkinson's disease; *R*, right.

P^{G} *P*-value of the group effect; P^{S} *P*-value of the sex effect; P^{GS} *P*-value of the group by sex interaction effect.

Supplementary Table 2 Comparisons between PD males and PD females in regions showing significant group by sex interactions in cortical thickness parcellations

	PD males	PD females	PD females > PD males		
			t	***P*-value**	***d***
Global					
L mean thickness	2.40±0.11	2.41±0.11		—	
R mean thickness	2.40±0.10	2.42±0.11	2.153	0.040	0.280
Frontal					
L caudal middle frontal	2.46±0.14	2.51±0.15	2.831	0.019	0.395
L precentral	2.51±0.15	2.54±0.16		—	
L superior frontal	2.60±0.13	2.64±0.13	3.036	0.019	0.423
R caudal middle frontal	2.43±0.14	2.49±0.15	3.040	0.019	0.437
R pars orbitalis	2.54±0.17	2.61±0.16	3.331	0.019	0.473
R precentral	2.46±0.17	2.49±0.16	2.327	0.022	0.315
R superior frontal	2.56±0.13	2.62±0.14	3.612	0.019	0.511
Temporal					
L inferior temporal	2.71±0.16	2.68±0.14		—	
L middle temporal	2.73±0.16	2.70±0.16		—	
L transverse temporal	2.25±0.23	2.28±0.22		—	
R entorhinal	3.50±0.34	3.52±0.37		—	
R middle temporal	2.77±0.15	2.78±0.15		—	
Parietal					
L inferior parietal	2.33±0.14	2.36±0.14	2.315	0.040	0.308
L postcentral	2.03±0.13	2.05±0.13		—	
L precuneus	2.30±0.14	2.30±0.15		—	
L superior parietal	2.13±0.l4	2.15±0.14		—	
L supramarginal	2.43±0.14	2.46±0.15	2.308	0.040	0.311
R inferior parietal	2.39±0.14	2.41±0.15	2.131	0.048	0.273
R paracentral	2.37±0.14	2.38±0.15		—	
R precuneus	2.30±0.15	2.31±0.14		—	
R superior parietal	2.12±0.14	2.13±0.14		—	
Occipital					
L fusiform	2.65±0.14	2.66±0.13		—	
L lateral occipital	2.12±0.13	2.14±0.12		—	
L lingual	1.96±0.11	1.94±0.10		—	
R fusiform	2.67±0.15	2.66±0.14		—	
R lateral occipital	2.21±0.14	2.21±0.14		—	
R lingual	1.99±0.10	1.98±0.12		—	
Limbic					
R isthmus cingulate	2.31±0.21	2.34±0.23		—	
R posterior cingulate	2.38±0.16	2.43±0.17	2.881	0.021	0.400

Cortical thickness data in mm are presented by groups as mean ± SD. General linear model including age, years of education and age of onset as covariates and Monte Carlo permutation testing with 999 iterations followed by FDR correction (*P*-value ≤ 0.05) was used to compute between sexes comparisons. Abbreviations: *L*, left; *PD*, Parkinson’s disease; *R*, right.

Supplementary Table 3 Comparisons between PD males and PD females adjusting for BPF in regions showing significant sex differences

	PD males	PD females	PD females > PD males		
			t	***P*-value**	***d***
Global					
R mean thickness	2.40±0.10	2.42±0.11	2.106	0.023	0.259
Frontal					
L caudal middle frontal	2.46±0.14	2.51±0.15	2.797	0.009	0.389
L superior frontal	2.60±0.13	2.64±0.13	2.998	0.003	0.402
R caudal middle frontal	2.43±0.14	2.49±0.15	3.003	0.009	0.409
R pars orbitalis	2.54±0.17	2.61±0.16	3.335	0.003	0.489
R precentral	2.46±0.17	2.49±0.16	2.284	0.017	0.311
R superior frontal	2.56±0.13	2.62±0.14	3.578	0.003	0.487
Parietal					
L inferior parietal	2.33±0.14	2.36±0.14	2.282	0.023	0.307
L supramarginal	2.43±0.14	2.46±0.15	2.279	0.022	0.306
R inferior parietal	2.39±0.14	2.41±0.15	2.089	0.030	0.262
Limbic					
R posterior cingulate	2.38±0.16	2.43±0.17	2.842	0.003	0.366

Cortical thickness data in mm are presented by groups as mean ± SD. General linear model including age, years of education, age of onset, and brain parenchymal fraction as covariates and Monte Carlo permutation testing with 999 iterations followed by FDR correction (*P*-value ≤ 0.05) was used to compute between sexes comparisons. Abbreviations: *BPF*, brain parenchymal fraction; *L*, left; *PD*, Parkinson's disease; *R*, right.

Supplementary Table 4 Resulting models from regression analyses including age of onset for prediction of mean cortical thickness in regions showing sex differences

	**PD males**		**PD females**	
	Variables	t-stat (*P*-value)	Variables	t-stat (*P*-value)
L caudal middle frontal	Age	**-3.748 (≤0.001)**	Age	**-2.689 (0.009)**
	Age of onset	**2.678 (0.008)**		
	Education	-1.949 (0.053)		
L superior frontal	Age	**-3.728 (≤0.001)**	Age	**-3.103 (0.003)**
	Age of onset	**2.346 (0.020)**	Education	-1.431 (0.156)
R caudal middle frontal	Age	**-2.900 (0.004)**	Non-significant model	
	Age of onset	1.811 (0.072)		
R pars orbitalis	Age	**-3.016 (0.003)**	Age	**-2.637 (0.010)**
	Age of onset	**2.149 (0.034)**	Age of onset	**2.145 (0.035)**
	Education	-1.741 (0.084)	Education	-1.632 (0.107)
R precentral	Age	**-4.667 (≤0.001)**	Age	**-2.493 (0.015)**
	Age of onset	**2.164 (0.032)**		
R superior frontal	Age	**-3.055 (0.003)**	Age	**-2.751 (0.008)**
	Age of onset	**2.242 (0.027)**	Education	**-2.291 (0.025)**
L inferior parietal	Age	**-5.426 (≤0.001)**	Age	**-3.279 (0.002)**
	Age of onset	**3.486 (≤0.001)**	Age of onset	1.638 (0.106)
			Education	-1.652 (0.103)
L supramarginal	Age	**-4.848 (≤0.001)**	**Age**	**-3.190 (0.002)**
	Age of onset	**2.678 (0.008)**		
R inferior parietal	Age	**-6.176 (≤0.001)**	Age	**-3.518 (≤0.001)**
	Age of onset	**3.659 (≤0.001)**	Age of onset	1.738 (0.087)
R posterior cingulate	Age	**-3.581 (≤0.001)**	Age	**-2.914 (0.005)**
	Age of onset	**2.214 (0.029)**	Age of onset	1.592 (0.116)

Linear regression analyses with stepwise selection based on the Akaike information criterion (AIC) were applied separately in male and female patients for each region showing sex differences (response variable) with age, age of onset, and years of education introduced as explanatory variables. The selected variables for each model are shown, with statistically significant response variables in bold (*P*-value ≤ 0.05). Abbreviations: *L*, left; *PD*, Parkinson's disease; *R*, right.

Supplementary Table 5 Goodness of fit of the resulting models from regression analyses including age of onset for prediction of mean cortical thickness in regions showing sex differences

	**PD males**			**PD females**		
	Adjusted R^2	*F*	*P*-value	Adjusted R^2	*F*	*P*-value
L caudal middle frontal	0.088	5.345	0.002	0.078	7.233	0.009
L superior frontal	0.102	8.639	≤ 0.001	0.094	4.833	0.011
R caudal middle frontal	0.059	5.269	0.006	Non-significant model		
R pars orbitalis	0.054	3.551	0.016	0.073	2.935	0.039
R precentral	0.205	18.410	≤ 0.001	0.066	6.213	0.015
R superior frontal	0.059	5.033	0.008	0.089	4.617	0.013
L inferior parietal	0.200	17.920	≤ 0.001	0.131	4.715	0.005
L supramarginal	0.189	16.720	≤ 0.001	0.110	10.180	0.002
R inferior parietal	0.264	25.230	≤ 0.001	0.174	8.773	≤ 0.001
R posterior cingulate	0.095	8.117	≤ 0.001	0.109	5.528	0.006

Linear regression analyses with stepwise selection based on the Akaike information criterion (AIC) were applied separately in male and female patients for each region showing sex differences (response variable) with age, age of onset, and years of education introduced as explanatory variables. The goodness of fit, statistic and statistical significance for each resulting model are shown. Abbreviations: *L*, left; *PD*, Parkinson’s disease; *R*, right.

Supplementary Table 6 Regions showing significant sex by group interactions in subcortical gray matter volume segmentations

	HC males	PD males	HC females	PD females	*F*	P^{G}	P^{S}	P^{GS}	HC males > HC females			PD males > HC males			PD males > PD females			PD males > PD females[a]		
									t	*P*-value	*d*	*t*	*P*-value	*d*	*t*	*P*-value	*d*	*t*	*P*-value	*d*
L thalamus	6.53±0.60	7.00±0.78	6.73±0.45	6.70±0.67	5.819	0.015	0.010	0.011		—		3.720	0.002	0.581		—		1.664	0.045	0.211
R thalamus	6.44±0.58	6.83±0.72	6.64±0.46	6.51±0.58	5.171	0.015	0.010	0.011		—		3.365	0.002	0.494	2.129	0.032	0.255	2.138	0.032	0.257
R amygdala	1.69±0.22	1.64±0.23	1.56±0.25	1.52±0.23	5.021	0.019	0.015	0.011	2.417	0.016	0.487		—		2.690	0.020	0.339	2.651	0.024	0.335
Brainstem	21.02±1.98	22.03±2.03	20.76±1.75	21.18±2.02	5.588	0.015	0.010	0.011		—		2.721	0.004	0.460	2.068	0.032	0.287	2.038	0.035	0.284

Volumetric data in ml are presented by groups as mean±SD. General linear model including age and years of education as covariates and Monte Carlo permutation testing with 999 iterations followed by FDR correction (*P*-value ≤ 0.05) was used to compute group, sex, group by sex interaction effects, and post hoc pairwise comparisons. Abbreviations: *FDR*, false discovery rate; *HC*, healthy controls; *L*, left; *PD*, Parkinson's disease; *R*, right.

P^{G} *P*-value of the group effect; P^{S} *P*-value of the sex effect; P^{GS} *P*-value of the group by sex interaction effect.

[a] General linear mode including age of onset as an extra covariate.

Supplementary Table 7 Participants from each center

Center	PD males	PD females	HC males	HC females
Site 1	22	14	15	11
Site 2	50	36	14	15
Site 3	26	6	8	8
Site 4	38	19	6	9
**Total**	136	75	43	43

Abbreviations: *HC*, healthy controls; *PD*, Parkinson's disease.
