# Supplementary material for: A multi-site study on sex differences in cortical thickness in non-demented Parkinson’s disease
Source: NPJ Parkinsons Dis. 2024 Mar 23;10:69. doi: 10.1038/s41531-024-00686-2 (PMC10960793; doi:10.1038/s41531-024-00686-2)
Supplement: Supplementary file 2 — Related Manuscript File [file 41531_2024_686_MOESM2_ESM.pdf]

nature portfolio

Corresponding author(s): Barbara Segura

Last updated by author(s): Feb 28, 2024

Reporting Summary

Nature Portfolio wishes to improve the reproducibility of the work that we publish. This form provides structure for consistency and transparency in reporting. For further information on Nature Portfolio policies, see our Editorial Policies and the Editorial Policy Checklist.

Statistics

For all statistical analyses, confirm that the following items are present in the figure legend, table legend, main text, or Methods section.

n/a	Confirmed	
☐	☒	The exact sample size (*n*) for each experimental group/condition, given as a discrete number and unit of measurement
☐	☒	A statement on whether measurements were taken from distinct samples or whether the same sample was measured repeatedly
☐	☒	The statistical test(s) used AND whether they are one- or two-sided<br>*Only common tests should be described solely by name; describe more complex techniques in the Methods section.*
☐	☒	A description of all covariates tested
☐	☒	A description of any assumptions or corrections, such as tests of normality and adjustment for multiple comparisons
☐	☒	A full description of the statistical parameters including central tendency (e.g. means) or other basic estimates (e.g. regression coefficient) AND variation (e.g. standard deviation) or associated estimates of uncertainty (e.g. confidence intervals)
☐	☒	For null hypothesis testing, the test statistic (e.g. *F*, *t*, *r*) with confidence intervals, effect sizes, degrees of freedom and *P* value noted<br>*Give P values as exact values whenever suitable.*
☒	☐	For Bayesian analysis, information on the choice of priors and Markov chain Monte Carlo settings
☒	☐	For hierarchical and complex designs, identification of the appropriate level for tests and full reporting of outcomes
☐	☒	Estimates of effect sizes (e.g. Cohen's *d*, Pearson's *r*), indicating how they were calculated

Our web collection on statistics for biologists contains articles on many of the points above.

Software and code

Policy information about availability of computer code

Data collection	n/a
Data analysis	FreeSurfer v6.0.0, ComBat implementation in Matlab R2020b, SPSS version 27.0, Matlab R2020b, JASP version 0.14.3, using R version 4.1.2 with the RStudio version 2022.02.0 interface.

For manuscripts utilizing custom algorithms or software that are central to the research but not yet described in published literature, software must be made available to editors and reviewers. We strongly encourage code deposition in a community repository (e.g. GitHub). See the Nature Portfolio guidelines for submitting code & software for further information.

Data

Policy information about availability of data

All manuscripts must include a data availability statement. This statement should provide the following information, where applicable:

- Accession codes, unique identifiers, or web links for publicly available datasets
- A description of any restrictions on data availability
- For clinical datasets or third party data, please ensure that the statement adheres to our policy

The de-identified data that support the findings of this study are available on request from the corresponding author (BS). The data are not publicly available due to privacy or ethical restrictions

Research involving human participants, their data, or biological material

Policy information about studies with human participants or human data. See also policy information about sex, gender (identity/presentation), and sexual orientation and race, ethnicity and racism.

Reporting on sex and gender	Participants in both healthy control (HC) and Parkinson's Disease (PD) were grouped by sexes in accordance to the study aims.
Reporting on race, ethnicity, or other socially relevant groupings	n/a
Population characteristics	211 non-demented PD patients (64.45% males; mean age 65.58±8.44 years old; mean disease duration 6.42±5.11 years) and 86 healthy controls (50% males; mean age 65.49±9.33 years old).
Recruitment	We used multi-site MRI data from four research centers: The University of Deusto (Bilbao, Spain; Site 1), the University of Barcelona (Barcelona, Spain; Site 2), the Center of Addiction and Mental Health (CAMH; Toronto, Canada; Site 3), and the University of Cologne (Cologne, Germany; Site 4). The initial sample comprised 216 PD and 87 healthy control individuals, previously described in Monté-Rubio et al. The PD patients included in this sample fulfilled the UK PD Society Brain Bank diagnostic criteria for PD and were classified as non-demented according to the Level I for PD dementia diagnosis from the Movement Disorder Society Task Force on Dementia in Parkinson's Disease.<br><br>References:<br><br>Monte-Rubio, G. C. et al. Parameters from site classification to harmonize MRI clinical studies: Application to a multi-site Parkinson's disease dataset. Hum Brain Mapp 43, 3130–3142 (2022).
Ethics oversight	All participating sites received approval from an ethical standards committee prior to study initiation (i.e., Site 1, Clinical Research Ethics Committee of the Basque Country and Ethics Committee of the University of Deusto; Site 2, Ethics Committee of the University of Barcelona; Site 3, Ethics Committee of the Centre for Addiction and Mental Health; Site 4, Ethics Committee of the Medical Faculty of the University of Cologne), the study was conducted according to the guidelines of the Declaration of Helsinki, and all sites obtained written informed consent for research from all participants in the study.

Note that full information on the approval of the study protocol must also be provided in the manuscript.

Field-specific reporting

Please select the one below that is the best fit for your research. If you are not sure, read the appropriate sections before making your selection.

☒ Life sciences ☐ Behavioural & social sciences ☐ Ecological, evolutionary & environmental sciences

For a reference copy of the document with all sections, see nature.com/documents/nr-reporting-summary-flat.pdf

Life sciences study design

All studies must disclose on these points even when the disclosure is negative.

Sample size	The initial sample comprised 216 PD and 87 healthy control individuals, previously described in Monté-Rubio et al.<br><br>References:<br><br>Monte-Rubio, G. C. et al. Parameters from site classification to harmonize MRI clinical studies: Application to a multi-site Parkinson's disease dataset. Hum Brain Mapp 43, 3130–3142 (2022).
Data exclusions	After preprocessing, we excluded 5 PD and 1 HC due to segmentation problem. Our final sample comprised 211 PD and 86 HC volunteers: 136 PD males, 75 PD females, 43 HC males, and 43 HC females.
Replication	Data are available on request from the corresponding author (BS).
Randomization	We grouped participants by sexes. Sociodemographic and clinical variables were used as covariates as required.
Blinding	Blinding no necessary because no intervention was carried out.

Reporting for specific materials, systems and methods

We require information from authors about some types of materials, experimental systems and methods used in many studies. Here, indicate whether each material, system or method listed is relevant to your study. If you are not sure if a list item applies to your research, read the appropriate section before selecting a response.

Materials & experimental systems

n/a	Involved in the study
☒	☐ Antibodies
☒	☐ Eukaryotic cell lines
☒	☐ Palaeontology and archaeology
☒	☐ Animals and other organisms
☐	☒ Clinical data
☒	☐ Dual use research of concern
☒	☐ Plants

Methods

n/a	Involved in the study
☒	☐ ChIP-seq
☒	☐ Flow cytometry
☐	☒ MRI-based neuroimaging

Clinical data

Policy information about clinical studies

All manuscripts should comply with the ICMJE guidelines for publication of clinical research and a completed CONSORT checklist must be included with all submissions.

Clinical trial registration: n/a

Study protocol: n/a

Data collection: Data collection was managed by four research centers: The University of Deusto (Bilbao, Spain; Site 1), the University of Barcelona (Barcelona, Spain; Site 2), the Center of Addiction and Mental Health (CAMH; Toronto, Canada; Site 3), and the University of Cologne (Cologne, Germany; Site 4).

Outcomes: Primary outcomes: MRI measures [estimations of regional and global mean cortical thickness (CTh)], sociodemographic variables (age, sex, education), clinical variables (age of onset, disease duration).

Secondary outcomes: estimations of white matter hypointensities (WM-hypo), brain parenchymal fraction (BPF), subcortical gray matter volumes.

Plants

Seed stocks: n/a

Novel plant genotypes: n/a

Authentication: n/a

Magnetic resonance imaging

Experimental design

Design type: Structural MRI study.

Design specifications: n/a

Behavioral performance measures: n/a

Acquisition

Imaging type(s): T1-weighted.

Field strength: 3 Tesla.

Sequence & imaging parameters: Site 1: an MRI scanner Philips Achieva 3 T TX was used to obtain the images in a sagittal orientation. Repetition time (TR) = 7.4 ms, echo time (TE) = 3.4 ms, matrix size 228 ×218mm2; flip angle 9°, field of view (FOV) = 250mm, slice thickness 1.1mm, acquisition time = 4′55″, 300 slices, voxel size 0.98 ×0.98 ×0.60mm3.

Site 2: an 8-channel head coil SIEMENS MAGNETOM TrioTim syngo MR B19 3 T scanner (Siemens) was used to obtain

high-resolution three-dimensional (3D) T1-weighted images in a sagittal orientation. TR = 2,300ms, TE = 2.98ms, matrix size = 256 ×256mm2, flip angle 9°, FOV = 256mm, acquisition time = 7′48″, 240 slices, voxel size 1.0 ×1.0 ×1.0mm3.

Site 3: images were acquired through a General Electric Discovery MR750 3 T scanner, using a fast-spoiled gradient echo pulse sequence in a sagittal orientation. TR = 6.7 ms, TE = 3.0 ms, matrix size 256×256mm2, flip angle 8°, FOV = 230 mm, acquisition time = 4′16″, 200 slices, voxel size 0.89×0.89×0.9mm3.

Site 4: a PRISMA MAGNETOM 3 T scanner (Siemens) was used to obtain T1-weighted images in a sagittal orientation. TR = 2,300ms, TE = 2.32ms, matrix size 256×256mm2, flip angle = 8°, FOV = 230mm, acquisition time = 5′30″, 192 slices, voxel size 0.9 ×0.9 ×0.9mm3.

Area of acquisition: Whole brain.

Diffusion MRI: ☐ Used ☒ Not used

Preprocessing

Preprocessing software: Regional mean CTh estimations of the 68 cortical brain regions of the Desikan-Killiany atlas and left and right hemisphere mean CTh estimations were extracted after applying the automated processing pipeline and FreeSurfer v6.0.0 tools (https://surfer.nmr.mgh.harvard.edu/). The stream includes the parcellation of the cerebral cortex and its automated labeling.

We extracted WM-hypo)volume as a proxy of small vessel disease after applying a probabilistic labelling implementation in FreeSurfer v6.0.0 applied to T1-weighted images. We computed BPF as the ratio of gray matter plus white matter excluding ventricles (i.e., BrainSegNotVent from Freesurfer) to eTIV. We extracted subcortical gray matter volumes extracted using FreeSurfer v6.0.0 (i.e., in the thalamus, putamen, pallidum, caudate, hippocampus, amygdala, accumbens, and brainstem).

We applied the prior validated ComBat method to harmonize regional mean CTh values across sites using its implementation in Matlab R2020b and the parametric empirical Bayes framework.

Normalization: Specifications above (Preprocessing software section).

Normalization template: Specifications above (Preprocessing software section).

Noise and artifact removal: Specifications above (Preprocessing software section).

Volume censoring: Specifications above (Preprocessing software section).

Statistical modeling & inference

Model type and settings: Group and sex effects in sociodemographic variables were analyzed through two-way analysis of variance (ANOVA) models followed by least significant difference (LSD) tests. Sex differences in clinical variables were analyzed by independent samples t-tests. These analyses were performed using SPSS version 27.0.

We applied generalized linear models (GLM) with Monte Carlo permutation tests with 999 iterations using in-house methods written in Matlab R2020b to analyze group, sex, and group-by-sex interaction effects for the 68 regional and left and right automated mean CTh harmonized estimations. Then, we performed post hoc pairwise comparisons only for those regions showing statistically significant interactions, also using GLM models and Monte Carlo permutation tests with 999 iterations. Cohen's d effect sizes were computed for post hoc comparisons using JASP version 0.14.3. Clinical, sociodemographic, and MRI variables were used as covariates as required.

The statistical significance threshold was set at a two-tailed P-value ≤ 0.05.

Effect(s) tested: n/a

Specify type of analysis: ☐ Whole brain ☒ ROI-based ☐ Both

Anatomical location(s): Desikan-Killiany atlas and AsegAtlas

Statistic type for inference (See Eklund et al. 2016): n/a

Correction: False discovery rate (FDR) approach, through the Benjamini-Hochberg procedure.

Models & analysis

n/a	Involved in the study
☒	☐ Functional and/or effective connectivity
☒	☐ Graph analysis
☐	☒ Multivariate modeling or predictive analysis

Multivariate modeling and predictive analysis

Multiple linear regression analyses were conducted to explore the age, disease duration, and age of onset effects on regions showing sex differences in PD. As a response variable, each model included a mean CTh estimation showing statistically significant sex differences in PD and age, plus disease duration or age of onset as explanatory variables, as well as the sociodemographic covariates introduced in the previous GLM models (i.e. years of education). We tested the models separately for PD male and PD female patients. A stepwise model selection procedure by the Akaike information criterion (AIC) was applied to select the best-fitted model. Analyses were also performed using R version 4.1.2 with the RStudio version 2022.02.0 interface. The statistical significance threshold was set at a two-tailed P-value ≤ 0.05.
